# Combination of Anti-PD-1 Antibody, Anlotinib and Pegaspargase “Sandwich” With Radiotherapy in Localized Natural Killer/T Cell Lymphoma

**DOI:** 10.3389/fimmu.2022.766200

**Published:** 2022-02-14

**Authors:** Peng Sun, Yu Wang, Hang Yang, Cui Chen, Man Nie, Xiao-Qing Sun, Xiao-Hua He, Kang-Ming Huang, Jia-Jia Huang, Zhi-Ming Li

**Affiliations:** ^1^ Department of Medical Oncology, Sun Yat-Sen University Cancer Center, Guangzhou, China; ^2^ State Key Laboratory of Oncology in South China, Collaborative Innovation Center for Cancer Medicine, Guangzhou, China; ^3^ Department of Oncology, The First Affiliated Hospital, Sun Yat-Sen University, Guangzhou, China

**Keywords:** natural killer/T cell lymphoma, anlotinib, anti-PD-1 antibody, pegaspargase, radiotherapy

## Abstract

Asparaginase/pegaspargase containing regimens combined with radiotherapy are highly effective and considered the cornerstone of localized Natural killer/T-cell lymphoma (NKTL) treatment. However, these chemotherapy regimens inevitably cause relatively high incidence of treatment-related adverse events (TRAEs). Herein we retrospectively evaluated the efficacy and safety of the combined regimen of anti-PD-1 antibody, anlotinib and pegaspargase “sandwich” with radiotherapy in localized NKTL. Anti-PD-1 antibody and pegaspargase at 2500 U/m^2^ were administered on day 1, while anlotinib (12 mg once a day) was orally administered on days 1-14. The treatment was repeated every 3 weeks. All the eight patients included received 3 cycles of the regimen followed by radiotherapy and an additional 3 cycles. The overall response rate was 100%, and the complete response rate was 87.5%. With a median follow-up time of 35.5 months (range, 34.03-40.90 months), median PFS and OS times were not reached. The 3-year PFS and OS rates were 100% and 100%, respectively. All patients were alive at the last follow-up. No treatment-related death and no grade 4 TRAE was reported. No grade 3/4 hematological toxicity was detected, and half of the patients didn’t report any hematological toxicity. This study indicates that anti-PD-1 antibody combined with anlotinib and pegaspargase is a promising chemoradiotherapy regimen for localized NTKL, with mild toxicity and good tolerance.

## Introduction

Natural killer/T-cell lymphoma (NKTL) is a rare and distinct subtype of non-Hodgkin’s lymphoma (NHL), which predominantly occurs in East Asia and Latin America (1, 2). Appropriately 60% of NKTL cases are initially diagnosed as stage I/II disease in the upper aerodigestive tract (1). Radiotherapy has been widely used in localized NKTL, and the combined strategy of radiotherapy systematic treatment has greatly improved the clinical outcome of these patients (3, 4). Asparaginase and pegaspargase are key agents for NKTL treatment, and asparaginase/pegaspargase containing regimens (P-GEMOX, SMILE and DDGP) are highly effective in the combined treatment strategy (5–9). However, these chemotherapy regimens consist of multiple toxic agents and inevitably have some hematological toxicity and risk of infection (5–9). Meanwhile, severe toxicities would hamper the completion of combined radiotherapy and reduce patients’ medical compliance, which limits their usefulness in fragile patients and concurrent radiotherapy. Therefore, there is an unmet need for a more tolerable chemoradiotherapy regimen with comparable efficacy for localized NKTL.

Epstein–Barr virus (EBV) has been reported to increase the expression of programmed death-1 (PD-1) in various types of cancer, including NKTL (10–12). Therefore, it is reasonable to use anti-PD-1 antibody in NKTL patients. Several anti-PD-1 antibodies, including pembrolizumab, nivolumab and sintilimab, have been explored in refractory/relapsed NTKL, with impressive antitumoral effects in previously heavily treated patients (13–15). Such encouraging results in relapsed/refractory NKTL have provided the opportunity to explore anti-PD-1 antibodies in the frontline treatment of NKTL. Recently, Cai et al. explored the efficacy of anti-PD-1 antibodies with the P-GEMOX regimen in 9 advanced NKTL patients, with a complete response rate of 66.7% (16). The latter exploratory study indicated the potential promising therapeutic role of immune checkpoint inhibitors in first-line treatment of NKTL.

Currently, antiangiogenic treatments are widely used in multiple malignancies. Overexpression of genes related to angiogenesis have been observed in NKTL (17). In particular, overexpression of both vascular endothelial growth factor A (VEGF-A) and its receptor VEGFR2 has been detected in NKTL tissues and cell lines at the mRNA and protein levels (17). These findings provided a rationale to further explore the effect of antiangiogenic treatment in NKTL (17). Anlotinib is a small molecule tyrosine kinase inhibitor (TKI) targeting vascular endothelial growth factor receptor (VEGFR), fibroblast growth factor receptor (FGFR) and platelet-derived growth factor receptor (PDGFR), with promising antitumor effect in lung cancer and sarcoma (18, 19). Moreover, a recent study demonstrated anlotinib could potentiate the therapeutic effect of PD-1 blockade by optimizing antitumor innate immunity in lung cancer (20). In addition, the potential synergistic effects of immune checkpoint inhibitors and antiangiogenic agents have been reported in hepatocellular, cervical and lung cancers (21–25). However, data regarding the combination therapy of anti-PD-1 antibody and anti-angiogenesis agents in NKTL are scarce.

Taken together, herein we proposed a novel systematic combination regimen of anti-PD-1 antibody, anlotinib and pegaspargase “sandwich” with radiotherapy for localized NKTL and reported retrospective results of 8 patients in our institution.

## Materials and Methods

### Ethics Approval and Consent to Participate

All patients provided consent for the collection and processing of clinicopathological data. The study was approved by the Bioethics Committee of Sun Yat-Sen University Cancer Center for a retrospective analysis of the collected data and undertaken in accordance with the ethical standards of the World Medical Association’s Declaration of Helsinki.

### Patients and Treatment

Eligible patients met the following inclusion criteria: (1) aged 18 years and older; (2) histologically confirmed NKTL at the clinical stage of I-II by Ann Arbor staging system; (3) had at least one evaluable lesion; (4) without previous anti-tumor therapy including radiotherapy, chemotherapy, targeted therapy or stem cell transplantation; (5) Eastern Cooperative Oncology Group (ECOG) status 0-3; (6) adequate organ and bone marrow function. Patients those with active hemorrhage or at the risk of hemorrhage, with other types of malignancy, with uncontrolled hypertension, history of immunodeficiency, didn’t undergo radiotherapy, and with involvement of central nervous system were excluded.

The regimen was repeated every 3 weeks. Anti-PD-1 antibodies (pembrolizumab/nivolumab/sintilimab, detailed information was listed in **Table 2**) and pegaspargase 2500 U/m^2^ *via* intramuscular injection on day 1, while anlotinib (12 mg once a day) was orally administered on days 1-14. In order to guarantee patients’ performance status and to reduce treatment-related toxicity, “sandwich” radiotherapy was performed to cases with NKTL limited to the nasal cavity or nasopharynx. All patients included received 3 cycles of the regimen followed by intensity-modulated radiotherapy (IMRT: PTV-GTV 54-56Gy/25-26F, PTV-CTV1 50-50.7Gy/25-26F and PTV-CTV2 45Gy/25-26F) and an additional 3 cycles. No maintenance of anti-PD-1 antibody was prescribed to these patients.

### Treatment Evaluation and Toxicity

Physical examination, bone marrow aspiration and biopsy, routine laboratory tests, electrocardiography and echocardiography were performed at baseline. ^18^-fluorodeoxyglucose (^18^F-FDG) positron emission tomography-computed tomography (PET/CT) or enhanced contrast computed tomography (CT) were performed to identify the disease stage and to evaluate treatment response. In addition, baseline magnetic resonance imaging (MRI) was displayed in patients who planned to undergo radiotherapy. The plasma load of EBV DNA was detected every cycle in each participant. Treatment response was performed every two cycles and assessed by investigators per Revised Response Criteria for Malignant Lymphoma (26). Upon cessation of treatment, each patient was followed up every 3 months for the first two years and every 6 months after two years, with test of EBV DNA and CT scan. Treatment-related adverse events (TRAEs) were evaluated with the Common Terminology Criteria for Adverse Events (CTCAE) version 5.0 (27).

### Statistical Analyses

Overall survival (OS) was determined as the time from diagnosis to the date of death or last follow-up visit, and progression free survival (PFS) was assessed as the time from diagnosis to relapse, progression, death or last follow-up visit. Survival curves were obtained by the Kaplan-Meier method, and OS and PFS rates were estimated by the log-rank test. Statistical analysis was performed with Statistical Package for the Social Sciences (SPSS) version 22.0.

## Results

### Patient Characteristics

A total of 12 biopsy-proven and previously untreated NKTL cases diagnosed with stage I/II disease underwent combination therapy with anti-PD-1 antibody, anlotinib plus pegaspargase from September 2018 to May 2019 in our institution. Two patients diagnosed as colon NKTL without evaluable lesion after surgery and two patients who didn’t undergo radiotherapy were excluded. Finally, eight participants were included in this retrospective analysis, with a median age of 57 years (range, 35-70); 7 (87.5%) were male, and 6 patients (75%) had Ann Arbor stage II disease. According to the PINK-E scoring system, score 0 was identified in two patients (25%), score 1 in two patients (25%) and score 2 in four patients (50%), respectively. EBV DNA load could be detected at baseline in six (75%) patients, ranging from 0 to 3110 copies/mL (median 424.5 copies/mL). All the patients’ clinical characteristics are summarized in **Table 1**.

**Table 1:** Clinicopathologic features on presentation.

Age (years)	Gender	KPS	Ann Arbor Stage	PINK-E	Primary Site	B symptoms	LDH (U/L)	HBsAg/HBcAb	Baseline EBV load (copies/mL)
57	M	90	2	1	Nasal cavities, maxilla	Yes	193.9	Neg/Neg	284
65	M	90	2	2	Tonsils	No	131.7	Neg/Pos	0
70	M	90	2	2	Nasal cavities, sinuses	No	298.4	Neg/Neg	3110
54	M	90	2	0	Nasal cavities	No	172.6	Neg/Pos	0
57	M	90	2	1	Nasopharynx, nasal cavities	Yes	201.1	Pos/Pos	1780
35	F	90	1	0	Nasal cavity	No	193.3	Neg/Pos	226
67	M	70	1	2	Nasal cavity	No	180.7	Neg/Pos	565
37	M	80	2	2	Gum, oropharynx, lymph nodes	Yes	209.6	Neg/Pos	2720

### Efficacy and Survival

**Figure 1:**
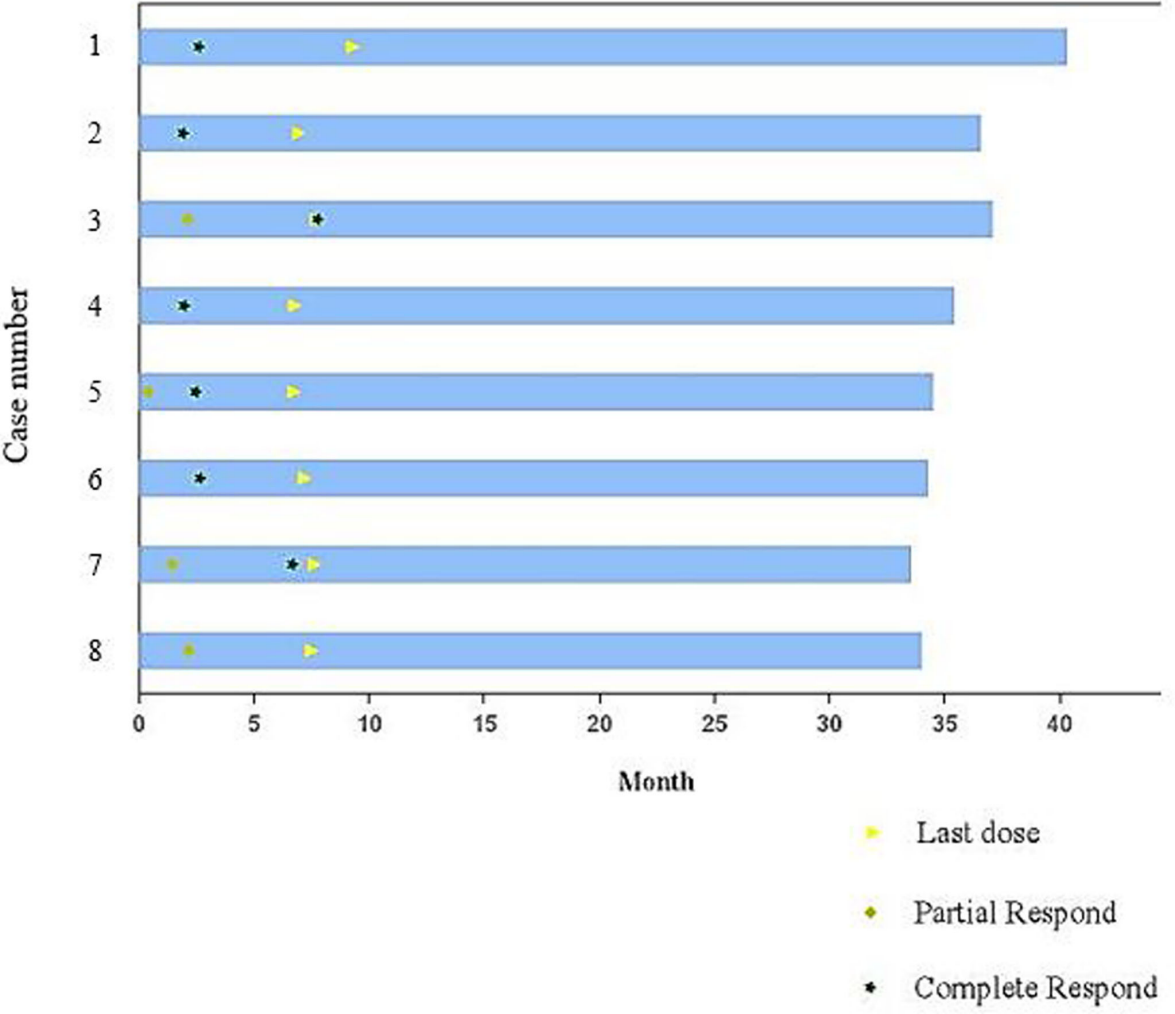
Durations of drug exposure.

All cases included were evaluable for treatment response. In all, the eight patients received 48 cycles of the regimen. After two cycles of combined treatment, the overall response rate (ORR) was 100%. Complete response (CR) was detected in 4 patients (4/8, 50%), and partial response (PR) was found in 4 patients (4/8, 50%). After six cycles of the regimen and “sandwich” radiotherapy, the ORR was still 100%, with CR in seven patients (87.5%) and PR in one patient (12.5%). All the eight patients were still in remission at the last follow-up (**Table 2** and **Figure 1**). The tumor lesion of Case 8 significantly contracted at the end of treatment, however, CT scan showed minimal residual tissues which could not be confirmed as CR and the remaining tissues were no change during the follow-up period.

**Table 2:** Therapies and outcomes of the 8 patients.

Case No.	PD-1 antibody/dose	Anlotinib	Pegaspargase	Response after 2nd cycle	Best Response	Duration of follow-up (months)
**1**	nivolumab/40mg	12mg D1-14	2500 IU/m^2^	CR	CR	40.9
**2**	pembrolizumab/100mg	12mg D1-14	2500 IU/m^2^	CR	CR	37.1
**3**	pembrolizumab/100mg	12mg D1-14	2500 IU/m^2^	PR	CR	37.63
**4**	pembrolizumab/100mg	12mg D1-14	2500 IU/m^2^	CR	CR	35.93
**5**	sintilimab/100mg	12mg D1-14	2500 IU/m^2^	PR	CR	35
**6**	sintilimab/200mg	12mg D1-14	2500 IU/m^2^	CR	CR	34.77
**7**	sintilimab/200mg	12mg D1-14	2500 IU/m^2^	PR	CR	34.03
**8**	sintilimab/200mg	12mg D1-14	2500 IU/m2	PR	PR	34.5

With a median follow-up time of 35.5 months (range, 34.03-40.90 months), the median PFS and OS times were not reached. The 3-year PFS and OS rates were 100% and 100%, respectively. All patients were alive at the last follow-up (10^th^ January 2022).

### Toxicity

All the patients had available toxicity data for safety analysis. No treatment-related death was reported. **Table 3** lists the detailed toxicity data. All the participants reported at least one TRAE. The most common TRAEs were hypoalbuminemia (100%), increased transaminases (100%), nausea (87.5%) and anorexia (87.5%). No TRAEs higher than grade 3 were observed. No grade 3/4 hematological toxicity was detected, and four patients (50%) didn’t report any hematological toxicity during the treatment course. Transfusion-related fever due to anti-PD-1 antibody administration was not found. Most of the non-hematological TRAEs were related to pegaspargase. Only one grade 3/4 non-hematological toxicity event was reported. Case 3 developed grade 3 hyperbilirubinemia after two cycles of the regimen and improved with medication of oral ademetionine. Neither grade 3/4 immune-related toxicity nor antiangiogenic toxicity was demonstrated. The most common anlotinib–related AEs were hypertension (25%), proteinuria (25%), hand-foot syndrome (25%). Only one immune-related AE (grade 1 hypothyroidism; Case 6) was reported without symptoms, and no medication was given. All the TRAEs were resolved with adequate treatment. No dose reduction or treatment discontinuation of any agent of the regimen was reported. Furthermore, no radiotherapy delay caused by TRAEs was found.

**Table 3:** Toxicities.

Toxicity	Grade (No., %)			
	1	2	3	4
**Hematological**				
Neutropenia	2 (25)	0	0	0
Anemia	2 (25)	1 (12.5)	0	0
Thrombocytopenia	1 (12.5)	0	0	0
**Non-Hematological**				
Nausea	4 (50)	3 (37.5)	0	0
Vomiting	3 (37.5)	0	0	0
Anorexia	4 (50)	3 (37.5)	0	0
Increased transaminases	5 (62.5)	3 (37.5)	0	0
Hyperbilirubinemia	2 (25)	2 (25)	1 (12.5)	0
Hyperglycemia	2 (25)	4 (50)	0	0
Hypoalbuminemia	4 (50)	4 (50)	0	0
Decreased fibrinogen	2 (25)	0	0	0
Hypertension	2 (25)	0	0	0
Hemorrhage	0	0	0	0
Proteinuria	1 (12.5)	1 (12.5)	0	0
Thrombosis	0	0	0	0
Rash	0	1 (12.5)	0	0
Hand-foot syndrome	2 (25)	0	0	0
Mucositis	5 (62.5)	1 (12.5)	0	0
Hypothyroidism	1 (12.5)	0	0	0
Increased amylase	0	0	0	0
Increased lipase	1 (12.5)	0	0	0
Diarrhea	0	0	0	0
Oral pain	4 (50)	1 (12.5)	0	0
Fatigue	5 (62.5)	0	0	0

## Discussion

To the best of our knowledge, this is the first study to report the efficacy and safety of anti-PD-1 antibody-based regimen in the frontline treatment of localized NKTL. The results suggested that the novel triple-agent regimen (anti-PD-1 antibody, anlotinib and pegaspargase) could be a highly active combination for untreated patients with localized NKTL. Moreover, this regimen had mild toxicity and showed a favorable safety profile when “sandwich” with radiotherapy, suggesting it as a feasible and promising treatment approach in the clinical setting.

Conventional anthracyclines-containing regimens have no favorable efficacy in NKTL due to frequent expression of P-gp (28), which could be partly overcome by L-asparaginase and pegaspargase (5–9). In the past decade, L-asparaginase and pegaspargase have been established as cornerstone in the therapy of NKTL patients, and several L-asparaginase and pegaspargase combining regimens have been explored and recommended as standard of care for NKTL (5–9). Pegaspargase is more feasible and has relatively lower risk of allergy compared with L-asparaginase and is therefore widely used in our institution (29). Thus, we consider pegaspargase an indispensable and cornerstone drug in the frontline treatment of localized NKTL.

Recent studies have demonstrated that anti-PD-1 antibody is an effective and revolutionary salvage treatment in relapsed/refractory NKTL. In our study, three distinct anti-PD-1 antibodies were used, all of which showed remarkable efficacy in relapsed/refractory NKTL. In 2017, Kwong et al. reported seven relapsed/refractory NKTL patients who all responded to pembrolizumab (5 CR and 2 PR) for the first time (13). Li et al. then demonstrated that pembrolizumab is highly effective in relapsed/refractory NKTL, with an ORR of 57% (15). Low dose nivolumab was also found to be effective in relapsed/refractory NKTL by Chan et al. (14). Sintilimab was reported to achieve an ORR of 67.9% and a 1-year OS of 82.1% in relapsed/refractory NKTL in the Orient-4 study (30). Nevertheless, anti-PD-1 antibody has also shown encouraging antitumor activity in the frontline therapy of NKTL (16).

Currently, anti-PD-1 antibody plus antiangiogenic tyrosine kinase inhibitor (TKI) has been explored in a variety of clinical trials of multiple solid tumors, demonstrating impressive synergistic antitumor effects (21–25). Vascular endothelial growth factor (VEGF) has been found to play an important role in systemic and local immunosuppression in tumor models (31). Increased levels of VEGF in tumor microenvironment could change the expression of adhesion molecules to directly inhibit the function of T cell, prevent T-cell activation, reduce the T cell-mediated anticancer immune response, and promote the recruitment and proliferation of immunosuppressive cells (31, 32). As a multitarget antiangiogenic TKI, anlotinib could firstly inhibit the VEGFR to normalize the tumor vessels, reducing tissue hypoxia and enhancing the delivery of other antitumor agents such as immune checkpoint inhibitor (32). Secondly, monotherapy of anlotinib was found to upregulate the expression of PD-L1 in the tumor microenvironment (TME), which could increase the antitumor effect of anti-PD-1 antibody (31, 32). Finally, anlotinib was identified to reprogram the immunosuppressive TME into an immunostimulatory TME by enhancing the infiltration of immune effector cells and the antigen presentation function (20). In addition, TME vessel normalization could conversely be promoted by immune checkpoint inhibitors (33). Collectively, strong reasons exist to support the multidimensional synergetic effects of anlotinib combined with anti-PD-1 antibodies. In a series of prospective and observational studies, the encouraging efficacy data of anti-PD-1 antibody plus anlotinib have been reported (22, 34–36). In this study, interim analysis after 2 cycles of treatment showed a CR rate (CRR) of 50% and an ORR of 100%, indicating that this regimen could induce a rapid remission in NKTL. Notably, the CRR increased to 87.5% when “sandwich” with radiotherapy. L-asparaginase/pegaspargase containing cytotoxic regimens, including SMILE, AspaMetDex and P-GEMOX/GELOX, have been previously explored in untreated and relapsed/refractory NKTL patients, exhibiting ORRs of 78%-96% and CRRs of 45%-74% (5–9, 29, 37). When concurrently, sequentially or “sandwich” combined with radiation, the CRRs of these regimens could be further increased to 70%-80%. Based on these findings, we suggested that our novel regimen and traditional cytotoxic regimens have comparable efficacies.

The combination of immune checkpoint inhibitors (ICI) and radiotherapy has recently been recognized as a breakthrough therapy in multiple malignancies. Beside the direct disruption of chemical bonds within the bases in the DNA of tumor cells, radiotherapy-induced abscopal effect, such as the reconstruction of tumor immune microenvironment, could play an important role in antitumor effects (38, 39). Radiotherapy could induce immunogenic cell death (ICD) to release new tumor antigens, upregulate MHCI molecules, activate dendritic cells, and reduce the abundance of immunosuppressive cells (38, 39). Accumulating evidence, both preclinical and clinical, indicates that anti-PD-1 antibodies combined with radiotherapy have synergistic effects in lung cancer, pancreatic cancer, prostate cancer and melanoma (38, 40, 41). Considering that radiotherapy remains the cornerstone of localized NKTL, anti-PD-1 antibody might show a potential synergetic effect when combined with radiotherapy. In this study, three patients who obtained PR in interim analysis ultimately achieved CR after radiotherapy. These promising data suggest that prospective studies are warranted to confirm the potential benefits and safety of the combination of radiotherapy and anti-PD-1 antibody-containing regimens in localized NKTL.

The major superior feature of our regimen is the encouraging safety profile, which is consistent with known safety profiles of anti-PD-1 antibodies, anlotinib or pegaspargase alone. No new safety signal was reported. Generally, grade 3/4 TRAEs were identified in only one patient (12.5%), whereas the incidence rates of grade 3/4 TRAEs were generally above 70% for traditional cytotoxic regimens (5–9, 29, 37). In previous cytotoxic regimens, the incidence of grade 3/4 hematological toxicity was generally above 50%, which was the major concern of scheduled radiotherapy. In this study, half of the participants didn’t suffer from any grade hematological toxicity, and no grade 3/4 hematological toxicity was reported. Meanwhile, immune-related AE and antiangiogenic AE were also mild. All TRAEs were alleviated within 3 months after the last dose. No dose reduction, treatment discontinuation or radiotherapy delay was reported due to TRAEs. According to the safety data, our regimen is a feasible and promising chemoradiotherapy regimen which can be well tolerated in fragile and elderly patients.

There were several limitations in this study. First, this study was limited by its nonrandomized nature with a relatively small sample size. Secondly, efficacy analyses were preliminary, and long-term follow-up is still needed. Finally, we could not carry out prognostic analysis of biomarkers in this study. To overcome these shortcomings, a prospective, single arm, phase II trial evaluating the safety and efficacy of sintilimab, anlotinib and pegaspargase for localized NKTL has been initiated in multiple centers (NCT03936452).

In conclusion, these preliminary findings indicate that anti-PD-1 antibody combined with anlotinib and pegaspargase is a promising regimen “sandwich” with radiotherapy for localized NTKL, with encouraging efficacy, mild toxicity and good tolerance. Future prospective studies with large sample sizes could provide more accurate data on the efficacy and safety of this regimen and could further identify predictive biomarkers of this novel therapy in NKTL patients.

## Data Availability Statement

The datasets presented in this study can be found in online repositories. The names of the repository/repositories and accession number(s) can be found below: https://www.researchdata.org.cn (RDDA2021002103).

## Ethics Statement

The studies involving human participants were reviewed and approved by The Bioethics Committee of Sun Yat-Sen University Cancer Center. The patients/participants provided their written informed consent to participate in this study.

## Author Contributions

PS, YW, and CC collected clinical data and drafted the manuscript. PS and Z-ML participated in the design of the study. MN, X-HH, and X-QS performed the statistical analysis. Z-ML and J-JH conceived the study and participated in its design and coordination and helped draft the manuscript. PS, YW, HY and CC were co-first authors. All authors contributed to the article and approved the submitted version.

## Funding

This work was supported by grants from National Science and Technology Major Project (nos. 2018ZX09734003), the National Natural Science Foundation of China (nos.81872902, 82073917, 82103579, 82104273), the Youth Funds of the Basic and Applied Basic Research Foundation of Guangdong Province (No. 2020A1515110089), and the Sun Yat-Sen University Cancer Center Clinical Research 308 Program (nos. 2014-fxy-106 and 2016-fxy-079).

## Conflict of Interest

The authors declare that the research was conducted in the absence of any commercial or financial relationships that could be construed as a potential conflict of interest.

## Publisher’s Note

All claims expressed in this article are solely those of the authors and do not necessarily represent those of their affiliated organizations, or those of the publisher, the editors and the reviewers. Any product that may be evaluated in this article, or claim that may be made by its manufacturer, is not guaranteed or endorsed by the publisher.
